# Creatinine-Based Definition of Kidney Disease in the Charlson Comorbidity Index May Underestimate Prognosis in Males Compared to an Estimated Glomerular Filtration Rate Definition

**DOI:** 10.3390/jcm13041007

**Published:** 2024-02-09

**Authors:** Andy K. H. Lim, Peter G. Kerr

**Affiliations:** 1 Department of General Medicine, Monash Health, Clayton, VIC 3168, Australia; 2 Department of Nephrology, Monash Health, Clayton, VIC 3168, Australia; peter.kerr@monash.edu; 3 Department of Medicine, School of Clinical Sciences, Monash University, Clayton, VIC 3168, Australia

**Keywords:** Charlson comorbidity index, estimated glomerular filtration rate, chronic kidney disease, serum creatinine, kidney function, prognosis, public health, epidemiology

## Abstract

(1) **Background**: The Charlson comorbidity index allocates two points for chronic kidney disease (CKD) if serum creatinine is above 3.0 mg/dL (270 µmol/L). However, contemporary CKD staging is based on the estimated glomerular filtration rate (eGFR) derived from population-based equations. The aim of this study was to determine the correlation between eGFR and the creatinine threshold of the Charlson comorbidity index for defining CKD. (2) **Methods**: We conducted a cross-sectional study of 664 patients with established CKD attending general nephrology clinics over 6 months. Dialysis patients and kidney transplant recipients were excluded. (3) **Results**: The median age was 68 years, and 58% of the participants were male. By modeling with fractional polynomial regression, we estimated that a creatinine of 270 µmol/L corresponded with an eGFR of 14.8 mL/min/1.73 m^2^ for females and 19.4 mL/min/m^2^ for males. We also estimated that an eGFR of 15 mL/min/1.73 m^2^ (threshold which defines Stage 5 CKD) corresponded to a serum creatinine of 275 µmol/L for females and 342 µmol/L for males. After applying these sex-specific creatinine thresholds, 39% of males and 3% of females in our CKD study population who scored points for CKD in the Charlson comorbidity index had not yet reached Stage 5 CKD. (4) **Conclusions**: There is a significant difference in the creatinine threshold to define Stage 5 CKD between males and females, with a bias for greater allocation of Charlson index points for CKD to males despite similar eGFR levels between the sexes. Further research could examine if replacing creatinine with eGFR improves the performance of the Charlson comorbidity index as a prognostic tool.

## 1. Introduction

The Charlson comorbidity index was developed in 1987 by Charlson and her colleagues to utilize medical comorbidities to determine prognosis [1]. It uses a weighted measure of 19 items corresponding to medical chronic disease conditions to provide an assessment of mortality risk, including 1-year posthospitalization mortality. The Charlson index is used by clinicians to assist in planning treatment and shared decision making. It is the most widely used prognostic tool in clinical medicine and has been validated in numerous populations in both patients with and without cancer. Since the original version was developed, the age-adjusted Charlson index (additional 1 point for each decade over age 40 years, to a maximum of 4 points) with 17 items has been the most widely used adaptation and provides long-term prognosis with 10-year estimates of mortality. In a review by Charlson et al., the age-adjusted Charlson index was used to predict long-term mortality in patients with colorectal, gastric, pancreatic, prostate, ovarian, endometrial, lung, and renal cancer [2]. In nononcology patients, the age-adjusted Charlson index has been a predictor of survival in patients with hip fractures [3], hip arthroplasty [4], and general surgery patients [5].

One of the comorbidities included in the Charlson index is moderate to severe chronic kidney disease (CKD), defined as any of the following: (1) receiving dialysis treatment, (2) post kidney transplantation, or (3) a serum creatinine > 3.0 mg/dL (270 µmol/L). The presence of CKD contributes two points to the Charlson index. The Charlson index has been shown to predict mortality in incident hemodialysis patients [6,7] and incident peritoneal dialysis patients [8]. It has also been shown to be associated with 30-day unplanned readmissions in patients on maintenance hemodialysis [9]. To understand the potential impact of advanced CKD on prognosis based on the Charlson index, consider a patient aged 60–69 years, where the presence of advanced CKD in the absence of other comorbidities reduces a patient’s predicted 10-year survival from 90% to 53%. To our knowledge, there is little published data on patients with nondialysis CKD. Furthermore, serum creatinine reference ranges are different between males and females, and a single threshold to define advanced CKD may impact males and females differently.

The diagnosis, definition, and staging of CKD have evolved since the Charlson index was initially established. The estimated glomerular filtration rate (eGFR) is extensively used for the diagnosis of CKD and to determine CKD staging [10] and is routinely reported by most laboratories. The eGFR formulae were determined from population studies. In adults, the most widely used equation is the Chronic Kidney Disease Epidemiology Collaboration (CKD-EPI) equation, introduced in 2009 [11] with a subsequent revision in 2021 which included the removal of the race modifier [12]. There are currently no studies which have examined the correlation between eGFR and the Charlson index definition of CKD. Unlike the Charlson index, which does not factor in patient sex, the eGFR equations are modeled from population studies using sex-specific data. With the widespread acceptance of eGFR in CKD research and clinical practice, we were interested in determining what level of eGFR and contemporary staging of CKD would correspond to a positive CKD status defined by the Charlson index in clinical practice. Furthermore, we wanted to establish the difference in the proportion of patients who score points for CKD based on the traditional creatinine threshold compared to eGFR-defined kidney failure (Stage 5 CKD, eGFR < 15 mL/min/1.73 m^2^) and thus determine the potential impact on prognosis predicted by the Charlson index when using eGFR rather than the traditional criteria based on serum creatinine.

The aims of this study were to (1) define the threshold of eGFR corresponding to a positive Charlson CKD status in a real-world population of CKD patients while estimating the serum creatinine equivalent to an eGFR corresponding to Stage 5 CKD (eGFR < 15 mL/min/1.73 m^2^) and (2) to determine the magnitude of the difference in prognosis predicted by the Charlson index by comparing eGFR-defined kidney failure and the original fixed threshold creatinine level of 270 µmol/L.

## 2. Methods

### 2.1. Study Design and Patient Selection

We conducted a cross-sectional study of patients who attended two general nephrology outpatient clinics at a tertiary referral center (Dandenong Hospital and Monash Medical Centre, at Monash Health) in a 6-month period between March 2023 and August 2023. All adult patients (≥18 years) who attended these clinics were eligible for inclusion. If there was more than one attendance during this period, we only collected data from the latest review to avoid duplication. Patients were excluded if they were (1) receiving dialysis treatment, (2) hospitalized with acute kidney injury within the last 3 months, (3) new referrals undergoing assessment where CKD has not been established, and (4) patients who did not have biochemistry results available within the review period. The general nephrology clinics included a very limited number of patients with vasculitis or lupus nephritis, as there are specific clinics that manage these patients.

### 2.2. Chronic Kidney Disease

CKD was defined using Kidney Disease Improving Global Outcomes (KDIGO) criteria based on kidney damage or reduced kidney function [13]. The estimated glomerular filtration rate (eGFR) reported by laboratories using the CKD-EPI equation was used to stage CKD. A normal filtration rate is 90–100 mL/min. All Australian laboratories report the body surface area indexed eGFR values to 90 mL/min/1.73 m^2^, and some may report actual values above this, and others as >90 mL/min/1.73 m^2^. In the latter situation, we used the CKD-EPI equation calculator to derive the actual estimate ourselves [14]. Albuminuria was classified using the spot urine albumin–creatinine ratio, as normal (<3.4 mg/mmol), mild–moderate (3.4–34 mg/mmol), or severe (>34 mg/mmol). The etiology of CKD was categorized based on a documented clinician assessment. To keep categories mutually exclusive, we only accepted one primary diagnosis, which was considered the dominant cause of CKD. Disputes were resolved by discussion between investigators.

### 2.3. Charlson Comorbidity Index and Other Definitions

We identified all comorbidities from the medical records to determine the age-adjusted Charlson index. An online calculator was used to derive the Charlson index score for each patient [15]. The details of these items and weighted scores are shown in Appendix A Table A1. The Charlson index 10-year survival was estimated from the following equation:

$$0.983^{\left(e^{CCI\times 0.9}\right)},$$

where e = Euler’s constant (approximately 2.71828) and CCI is the Charlson index score. The results were multiplied by 100 and reported as a percentage. Patient age was determined at the time of the biochemistry sampling rather than clinic appointment or review date. Body mass index (BMI) classification was as follows: underweight (<18.5 kg/m^2^), healthy (18.5–24.9 kg/m^2^), overweight (25.0–29.9 kg/m^2^), obesity (30.0–39.9 kg/m^2^), morbid obesity (≥40.0 kg/m^2^).

### 2.4. Statistical Analysis

For descriptive statistics, we reported the mean and standard deviation (SD) for data with a normal distribution, or the median and interquartile range (IQR) for data with a skewed distribution. We used the chi-squared test for categorical data analysis. To compare means between males and females, we used the *t*-test. For a nonparametric test to compare distributions, we used the Mann–Whitney test. We used fractional polynomials to perform the regression of eGFR on serum creatinine. The aim was to find a model which most accurately predicts the mean eGFR given serum creatinine, and this method provides flexible parameterization for continuous variables. We used the best combination of 2 fractional polynomials (FP2 model, program default) to determine the best-fitting polynomial powers to allow for the nonlinear relationship between the variables. The deviance difference was used to compare 44 polynomial models tested. In general, the best model has the lowest deviance values. The graphs of the regression plots were inspected for satisfactory fit, and we examined the R^2^ (coefficient of determination) to assess how well the model regression line fits the data. All analyses were performed using STATA version 18 (StataCorp, College Station, TX, USA). A *p* < 0.05 was considered statistically significant.

## 3. Results

### 3.1. Patient Characteristics

During the study period, there were 1092 individual patients who were reviewed in the general nephrology clinics. There were 428 patients excluded due to long-term dialysis (*n* = 350), recent acute kidney injury (*n* = 17), lack of recent biochemistry (*n* = 51), and new referrals undergoing assessment (*n* = 10). Thus, a total of 664 patients with nondialysis CKD were included in the final analysis, and patient characteristics are summarized in Table 1.

The median age was 68 years, and 58.1% were male. Most patients had an elevated BMI, with 31% considered overweight, and another 42% were obese or morbidly obese. Diabetes was also highly prevalent, affecting almost half of patients. However, neither obesity nor diabetes was different in males compared to females. There was a higher prevalence of cardiovascular disease in males compared to females in terms of ischemic heart disease (*p* < 0.001) and peripheral vascular disease (*p* < 0.001). There was a higher prevalence of solid tumors in males compared to females (*p* = 0.001), which was predominantly due to prostate cancer (18 of 33 male patients). Thus, the mean Charlson index score was higher in males compared to females (mean difference = 0.92, *p* < 0.001).

### 3.2. Chronic Kidney Disease

**Figure 1:**
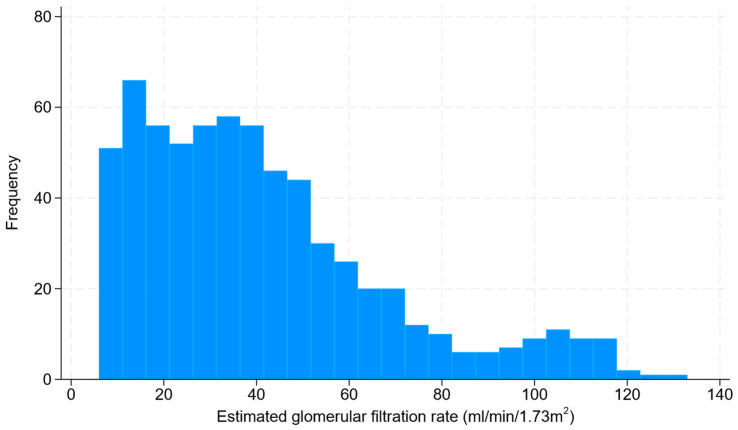
Histogram of the distribution of estimated glomerular filtration rate.

The distribution of eGFR was positively skewed and bimodal (Figure 1). While the majority of patients had an eGFR < 60 mL/min/1.73 m^2^, there was a small peak in the region of eGFR around 105 mL/min/1.73 m^2^, which was predominantly represented by patients with albuminuria and/or structural problems without a reduction in kidney function. Further details of CKD are summarized in Table 2.

Serum creatinine was overall higher in males compared to females (*p* < 0.001), and based on the Charlson index, there were more males than females who fulfilled the criterion for CKD with a serum creatinine > 270 µmol/L (27.2% vs. 13.0%, *p* < 0.0001). This contrasted the distribution of eGFR, which was not significantly difference between males and females (*p* = 0.21). There were more males with heavy albuminuria compared to females (*p* < 0.001), and females were more likely to be diagnosed with hypertensive nephrosclerosis.

### 3.3. Correlation between eGFR and Creatinine

**Figure 2:**
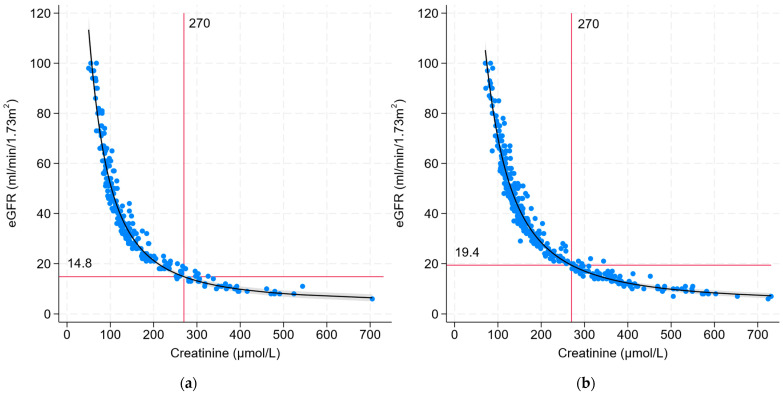
Correlation between the estimated glomerular filtration rate (eGFR) and serum creatinine by (**a**) female sex; (**b**) male sex. A polynomial regression line is fitted to the data independently for each sex, and the eGFR corresponding to a serum creatinine of 270 µmol/L (3.0 mg/dL) is shown by horizontal red lines (with the corresponding eGFR values of the intercept at the y-axis displayed above the line).

For the regression analysis, we limited our analysis to patients with an eGFR below 100 mL/min/1.73 m^2^ as the eGFR equations may be less accurate at higher estimates, evidenced by an increased dispersion of data points at higher eGFRs. Thus, 36 patients were excluded (female 67%; male 33%; mean age, 40 years; mean creatinine, 63 µmol/L). A graphical representation of the correlation between eGFR and serum creatinine for the remaining included patients is shown in Figure 2. Within the range of eGFR and creatinine of these patients, the data points remain fairly tight around the polynomial regression line, without a significant increase in the residuals (distance from the line of best fit). The R^2^ of 96% indicated an excellent fit of the predicted regression line to the observed data points. Patient sex was a statistically significant variable when included in the regression model of eGFR on creatinine. On average, and keeping creatinine constant, males had a 10.9 mL/min/1.73 m^2^ higher eGFR than females. Thus, we stratified the analysis and reported male and female regression results separately (Table 3).

For females, a serum creatinine of 270 µmol/L corresponded to an estimated eGFR of 14.8 mL/min/1.73 m^2^ (95% CI: 13.9–15.7 mL/min/1.73 m^2^). For males, this creatinine level corresponded with an eGFR of 19.4 mL/min/m^2^ (95% CI: 18.8–20.0 mL/min/1.73 m^2^). Therefore, the estimated difference in eGFR between the sexes was 4.6 mL/min/1.73 m^2^ at a creatinine threshold of 270 µmol/L.

Based on the regression model, an eGFR of 15 mL/min/1.73 m^2^ corresponded with a serum creatinine of 275 µmol/L (95% CI: 270–280 µmol/L) for females and 342 µmol/L (95% CI: 338–345 µmol/L) for males. Thus, the estimated difference in serum creatinine between the sexes was 67 µmol/L at an eGFR threshold of 15 mL/min/1.73 m^2^. If we applied these new serum creatinine thresholds to define Stage 5 CKD in our study population, 61% of male patients and 97% of female patients identified as Charlson index-positive CKD status would be classified as Stage 5 CKD, while the remaining would be classified as Stage 4 CKD.

### 3.4. Charlson Index and Survival Estimates by CKD Definition

In female patients, there was no significant change in mean Charlson index score when using the eGFR-based threshold definition of CKD failure (<15 mL/min/1.73 m^2^) compared to the fixed creatinine threshold (<270 µmol/L) definition of CKD (Charlson score, 4.02 vs. 4.06, *p* = 0.11). In contrast, for males, there was a significant change towards a lower mean Charlson score when using the eGFR criteria for CKD (4.50 vs. 4.78, *p* < 0.001). More specifically, 51 of 105 males (48.6%) and 6 of 36 (13.9%) females had a lower Charlson index score if we applied the eGFR definition of kidney failure (2 points less for CKD).

Table 4 summarizes the predicted 10-year survival estimates based on the Charlson index when comparing the fixed creatinine threshold definition of CKD (<270 µmol/L) and the eGFR-based threshold definition (<15 mL/min/1.73 m^2^). Overall, the mean 10-year estimated survival of males was lower than females. There was no significant difference in survival estimates for females when using the eGFR criteria to score points for CKD, but there was a significant difference for males. Table 5 summarizes the Charlson index-predicted 10-year survival estimates for patients who experienced a change in CKD status using the eGFR definition.

## 4. Discussion

In this epidemiological study, we examined the correlation between eGFR and the threshold of serum creatinine used to define CKD in the Charlson index. The Charlson index was developed prior to the introduction of estimating equations, which were correlated to measures of kidney function determined by radionuclide clearance. The strength of using eGFR is that it is derived from population studies, whereas unadjusted serum creatinine as a surrogate of kidney function does not take into consideration the patient’s age or sex. Thus, based on contemporary classification, we wondered if using eGFR rather than serum creatinine may improve the accuracy or precision of the Charlson index. As far as we are aware, there have been no published studies utilizing this approach.

We collected data from a population of patients attending general nephrology clinics as representative of a population with established CKD. We found that males had a higher comorbidity burden and Charlson index score than females, predominantly due to higher prevalences of cardiovascular disease, cancer, and CKD. We determined that a fixed creatinine threshold of 270 µmol/L (3.0 mg/dL) used to define CKD in the Charlson index has a different impact on males compared to females in relation to contemporary CKD staging based on eGFR. In the general CKD clinic population, we estimated that a creatinine threshold of 270 µmol/L (3.0 mg/dL) correlated to an average eGFR of 19.4 mL/min/1.73 m^2^ in males and 14.8 mL/min/1.73 m^2^ in females.

Next, we examined the hypothetical situation of using an eGFR of <15 mL/min/1.73 m^2^ to define CKD for scoring points in the Charlson index. This eGFR threshold defines kidney failure or Stage 5 CKD in contemporary classification [13]. We examined this impact in two ways. In the first approach (Section 3.3), we derived an estimate of serum creatinine which correlated to an eGFR of 15 mL/min/1.73 m^2^ from the regression models, which was 275 µmol/L and 342 µmol/L for females and males, respectively. By reclassifying patients based on this regression model, in terms of the derived sex-specific creatinine thresholds, we noted that 39% of male patients who scored points for CKD in the Charlson index did not have Stage 5 CKD. In contrast, only 3% of female patients who scored points for CKD in the Charlson index did not have Stage 5 CKD. In our second approach (Section 3.4), we directly applied a binary reclassification for Stage 5 CKD based on observed eGFR. By doing so, 49% of males and 14% of females who scored points for CKD on the creatinine definition would have 2 points less because their eGFRs were at 15 mL/min/1.73 m^2^ or above. Thus, it was reasonable to infer that the creatinine threshold of 270 µmol/L used in the current Charlson index reasonably approximates the threshold for Stage 5 CKD for females but contains a discrepancy for males. The main reason for the discrepancy between males and females is that the eGFR equation includes a sex modifier, which reduces a systematic bias associated with using a fixed serum creatinine threshold.

Although a single creatinine threshold provides simplicity of scoring the Charlson index, it overestimated advanced CKD in males compared to females. The implication of +2 points in the Charlson index on prognosis may not be trivial as it may impact the clinician assessment of survival. When we examined the 10-year survival estimates from the Charlson index and compared the eGFR and creatinine-based definitions for scoring CKD points, only the survival estimates for males showed a significant discrepancy (Section 3.4). For the 49% of male patients affected by this discrepancy, there was an average 27.1% difference in estimated 10-year survival. However, in the 14% of females with discrepant CKD scoring, a difference in the 10-year survival of 14.8% was not statistically significant (due to small numbers affected and lower Charlson index scores in general). Thus, there is a potential difference in prognostic estimates between males and females when we try to equalize the groups for kidney function based on eGFR.

Beyond the Charlson index, the importance of accurately defining CKD staging for prognostic reasons can be demonstrated by examining the mortality rates by CKD stage. In a large renal registry of over 1 million adults, the adjusted hazard ratio for death from any cause was 3.2 and 5.9 in patients with Stage 4 and Stage 5 CKD, respectively, compared to those with eGFR > 60 mL/min/1.73 m^2^ as the reference group [16]. Although the Charlson index has withstood the test of time in terms of its utility, many groups have modified the original index to suit their population of interest, such as a recalibration of the weighting system in liver transplantation [17] and peritoneal dialysis patients [18], or the exclusion of scores for other malignancies in patients with gastric cancer [19]. These studies have reported improved accuracy in predicting mortality with further refinement of the traditional Charlson index. Thus, it is sensible to reevaluate the performance of the Charlson index using eGFR rather than creatinine to define advanced CKD.

### 4.1. Strengths and Limitations

The strength of this study is the collection of data from a large number of established CKD patients, covering the spectrum of CKD stages, which allowed us to better model the relationship between eGFR and serum creatinine in a real-world CKD population. We were cautious to exclude patients with acute kidney injury, where the eGFR is not validated [20,21]. The limitations of this study include the paucity of patients with vasculitis and systemic lupus erythematosus, although we do not believe that it would have made a difference to the model estimates. This was a single-center study, and external validation would have been ideal to strengthen the findings. This study did not include dialysis patients, as serum creatinine and eGFR are meaningless in these patients. From a CKD staging perspective, there was also a high prevalence of obesity, which may have resulted in an underestimation of eGFR when standardized to a body surface area of 1.73 m^2^, and eGFR indexed to actual body surface area may be up to 50% higher [22]. Nonetheless, the CKD-EPI equation performed relatively well with acceptable bias in patients with eGFR < 60 mL/min/1.73 m^2^ and BMI < 40 kg/m^2^ [23]. Thus, it may be possible that the serum creatinine thresholds correlating to Stage 5 CKD may be different in a population with a different prevalence of obesity. In our study, the prevalence of obesity was similar in both sexes, so it was unlikely that the difference between the sexes was confounded by obesity. Lastly, our laboratories utilized the CKD-EPI equation, and the results may differ depending on the eGFR equations used. However, the CKD-EPI equation has been shown to be more accurate than the previously used Modification of Diet in Renal Disease (MDRD) equation, especially at higher eGFR values [24].

### 4.2. Implications for Clinical Practice

This was predominantly a hypothesis-generating study. It establishes a discrepancy in point scoring for CKD between males and females in the Charlson index when eGFR was used to hypothetically “match” the kidney function of males and females. Thus, for the same level of eGFR, prognosis appears to be underestimated for males relative to females. However, whether or not this discrepancy translates to an actual variation in prognosis requires validation with actual outcome data. Meanwhile, clinicians may consider interpreting the prognosis of males with creatinine 270–342 µmol/L with a grain of salt.

### 4.3. Research Recommendations

We suggest that future research studies examine the use of eGFR to define CKD to see if the performance of the Charlson index can be improved, and to align it with the contemporary classification of CKD. One option is to use the eGFR threshold of 15 mL/min/1.73 m^2^, and another is to raise the creatinine threshold for males to 342 µmol/L as the equivalent threshold of kidney function in females. One anticipated issue with using older data for this recalibration is the lack of isotope dilution mass spectrometry (IDMS) standardization of methods to measure serum creatinine. Serum creatinine measured without IDMS calibration may not be suitable for generating eGFR values from estimating equations such as CKD-EPI. Thus, new data sources with contemporary laboratory data may be needed to further evaluate this.

## 5. Conclusions

There is a significant difference in the creatinine threshold to define Stage 5 CKD between males and females, resulting in a bias for greater allocation of Charlson index points for CKD to males despite similar eGFR levels between the sexes. A serum creatinine level of 270 µmol/L closely approximates the threshold for Stage 5 CKD in females but not for males, resulting in a potential discrepancy in prognosis estimates for males.

## Figures and Tables

**Table 1:** Characteristics of patients with chronic kidney disease in general nephrology clinics.

Characteristics and Comorbidities	Total (*N* = 664)	Male (*n* = 386)	Female (*n* = 278)
Age in years, median (IQR)	68 (54–76)	69 (55–76)	65 (52–77)
Obesity, *n* (%) ^[1]^	242 (42.1)	136 (39.9)	106 (45.3)
Diabetes mellitus, *n* (%)	320 (48.1)	193 (50.0)	127 (45.7)
Ischemic heart disease, *n* (%) ^[2]^	158 (23.8)	119 (30.8)	39 (14.0)
Heart failure, *n* (%)	117 (17.6)	73 (18.9)	44 (15.8)
Chronic lung disease, *n* (%)	67 (10.1)	43 (11.1)	24 (8.6)
Chronic liver disease, *n* (%)	28 (4.2)	14 (3.6)	14 (5.0)
Stroke or transient ischemic attack, *n* (%)	64 (9.6)	42 (10.9)	22 (7.9)
Peripheral vascular disease, *n* (%) ^[2]^	48 (7.2)	41 (10.6)	7 (2.5)
Dementia, *n* (%)	13 (2.0)	8 (2.1)	5 (1.8)
Connective tissue disease, *n* (%)	36 (5.4)	19 (4.9)	17 (6.1)
Solid tumors, *n* (%) ^[2]^	40 (6.0)	33 (8.6)	7 (2.5)
Charlson chronic kidney disease, *n* (%) ^[2]^	141 (21.2)	105 (27.2)	36 (13.0)
Charlson index score, mean (SD) ^[2]^	4.3 (2.6)	4.7 (2.7)	3.7 (2.4)

^[1]^ missing = 89 (male 45, female 44). ^[2]^ Statistically significant difference between sexes.

**Table 2:** Chronic kidney disease staging and etiology in general nephrology clinic patients.

Chronic Kidney Disease Stage	Total (*N* = 664)	Male (*n* = 386)	Female (*n* = 278)
1. eGFR ≥ 90, *n* (%)	55 (8.3)	21 (5.4)	34 (12.2)
2. eGFR 60–89, *n* (%)	79 (11.9)	49 (12.7)	30 (10.8)
3. eGFR 30–59, *n* (%)	273 (41.1)	163 (42.1)	110 (39.6)
4. eGFR 15–29, *n* (%)	171 (25.8)	99 (25.7)	72 (25.9)
5. eGFR < 15, *n* (%)	86 (13.0)	54 (14.0)	32 (11.5)
Serum creatinine, median (IQR) ^[1]^	149 (112–236)	161 (125–280)	128 (92–190)
eGFR, median (IQR)	36 (21–54)	36 (20–52)	36 (22–57)
**Albuminuria**			
Normal (<3.4 mg/mmol), *n* (%)	188 (28.4)	86 (22.4)	102 (36.7)
Mild/moderate (3.4–34 mg/mmol), *n* (%)	204 (30.8)	121 (31.5)	83 (29.9)
Heavy/severe (>34 mg/mmol), *n* (%)	270 (40.8)	177 (46.1)	93 (33.5)
**Etiology**			
Diabetes mellitus, *n* (%)	244 (36.8)	154 (39.9)	90 (32.4)
Hypertensive nephrosclerosis, *n* (%)	119 (17.9)	52 (13.5)	67 (24.1)
Glomerulonephritis or glomerulopathy, *n* (%)	80 (12.1)	49 (12.7)	31 (11.2)
Renovascular disease, *n* (%)	69 (10.4)	43 (11.1)	26 (9.4)
Obstructive uropathy, *n* (%)	36 (5.4)	26 (6.7)	10 (3.6)
Polycystic kidney disease, *n* (%)	23 (3.5)	13 (3.4)	10 (3.6)
Interstitial nephritis/nephrotoxins, *n* (%)	14 (2.1)	8 (2.1)	6 (2.2)
Severe/recurrent acute kidney injury, *n* (%)	12 (1.8)	10 (2.6)	2 (0.7)
Nephrectomy/single kidney, *n* (%)	12 (1.8)	6 (1.6)	6 (2.2)
Congenital/reflux nephropathy, *n* (%)	11 (1.7)	6 (1.6)	5 (1.8)
Nephrocalcinosis, *n* (%)	9 (1.4)	1 (0.3)	8 (2.9)
Unclear or others, *n* (%)	35 (5.3)	18 (4.7)	17 (6.1)

^[1]^ Statistically significant difference between sexes.

**Table 3:** Fractional polynomial regression of eGFR on creatinine.

Model	Coefficients (95% CI)	Powers ^[1]^	R^2^
All patients, including sex variable	Creatinine_1 = 59.0 (57.5–60.5)	−2	0.94
	Creatinine_2 = 45.4 (42.4–48.4)	−2	
	Male sex = 10.9 (10.0–11.8)		
Female only	Creatinine_1 = 47.5 (45.5–49.5)	−2	0.96
	Creatinine_2 = 29.2 (25.6–32.7)	−2	
Male only	Creatinine_1 = 65.9 (64.3–67.5)	−2	0.96
	Creatinine_2 = 43.9 (38.4–49.3)	−2	

^[1]^ Best powers among 44 models fit.

**Table 4:** Ten-year survival estimates derived from the Charlson comorbidity index.

	Male (*n* = 374)	Female (*n* = 254)
	Mean % (95% CI)	Mean % (95% CI)
10-year survival (creatinine-based)	37.7 (33.7, 41.7)	48.2 (43.6, 52.9)
10-year survival (eGFR-based)	41.4 (37.4, 45.4)	48.6 (43.9, 53.2)
Difference in 10-year survival	3.7 (2.5, 4.9) *p* < 0.001	0.3 (0.1, 0.8) *p* = 0.13

**Table 5:** Ten-year survival estimates for patients with change in Charlson score using eGFR definition.

	Male (*n* = 51)	Female (*n* = 6)
	Mean % (95% CI)	Mean % (95% CI)
10-year survival (creatinine-based)	20.0 (11.3, 28.6)	63.7 (20.0, 100)
10-year survival (eGFR-based)	47.1 (36.8, 57.3)	78.4 (37.2, 100)
Difference in 10-year survival	27.1 (21.1, 33.0) *p* < 0.001	14.8 (−6.7, 36.3) *p* = 0.14

## Data Availability

Deidentified data may be available on reasonable request from the corresponding author, subject to approval by Monash Health Research Services.

## Appendix A

**Table A1:** Charlson comorbidity index score.

Comorbidity	Points
Age: 50–59 years	+1
Age: 60–69 years	+2
Age: 70–79 years	+3
Age: ≥80 years	+4
Prior myocardial infarction	+1
Congestive heart failure	+1
Peripheral vascular disease	+1
Stroke or transient ischemic attack	+1
Dementia	+1
Chronic pulmonary disease	+1
Connective tissue disease	+1
Peptic ulcer disease	+1
Liver disease: Mild	+1
Liver disease: Moderate–severe	+3
Diabetes: uncomplicated	+1
Diabetes: End-organ damage	+2
Hemiplegia or paraplegia	+2
Chronic kidney disease	+2
Solid tumor: Localized	+2
Solid tumor: Metastatic	+6
Leukemia	+2
Lymphoma	+2
AIDS	+6

Note: Age, liver disease, diabetes and solid tumor inputs are mutually exclusive (only one of the points categories can be selected for these variables).
